# Probabilistic Ecological Risk Assessment of Synthetic Musk Compounds in the Han River Estuary: Integrating Multi-Year Monitoring and Explainable Machine Learning

**DOI:** 10.3390/toxics14080718

**Published:** 2026-08-13

**Authors:** Jungmin Jo, Na Rae Choi, Eunjin Lee, Jae Won Yoo, Dong Sik Ahn, Ji Yi Lee, Yun Gyong Ahn

**Affiliations:** 1 Department of Environmental Science and Engineering, Ewha Womans University, Seoul 03760, Republic of Korea; jjm@ewha.ac.kr (J.J.); leeej1678@kbsi.re.kr (E.L.); 2 Department of Environmental Engineering, Kangwon National University, Chuncheon 24341, Republic of Korea; narae@kangwon.ac.kr; 3 Gangwon Particle Pollution Research and Management Center, Region of Korea, Kangwon National University, Chuncheon 24341, Republic of Korea; 4 Metropolitan Seoul Center, Korea Basic Science Institute (KBSI), University-Industry Cooperation Building, 150 Bugahyeon-ro, Seodaemun-gu, Seoul 03759, Republic of Korea; 5 Environmental Research Group, Korea Institute of Coastal Ecology, Bucheon 14449, Republic of Korea; jwyoo@coastkorea.com (J.W.Y.); ahndongsik@gmail.com (D.S.A.)

**Keywords:** synthetic musk compounds, emerging contaminants, ecological risk assessment, environmental exposure, XGBoost-SHAP, Monte Carlo simulation

## Abstract

Synthetic musk compounds (SMCs) are emerging contaminants frequently detected in aquatic environments due to their widespread use in personal care and household products. This study investigated the occurrence, environmental drivers, and ecological risks of twelve SMCs in the Han River estuary and adjacent coastal waters, Korea, using three years of monitoring data (2020–2022). A total of 222 water samples from 13 monitoring stations were analyzed by GC–MS. 1,3,4,6,7,8-hexahydro-4,6,6,7,8,8-hexamethylcyclopenta-(g)-2-benzopyran (HHCB) was the dominant compound, followed by musk ketone (MK). Total SMC concentrations ranged from non-detectable levels to 157.6 μg/L, with the highest concentrations consistently observed at wastewater treatment facilities. XGBoost-SHAP analysis identified total nitrogen (TN) as the most influential predictor of SMC occurrence. This association reflects the shared wastewater origin of the two rather than any causal link, so that TN serves as an indicator of wastewater influence rather than of SMC concentration itself. Ecological risks were evaluated using Monte Carlo-based probabilistic hazard quotient (HQ) analysis. HHCB and MK exhibited the highest risks, with mean HQ values exceeding 1.0 at wastewater treatment facilities, indicating potential ecological concern. In contrast, most coastal sites showed low risk levels. The observed spatial patterns identified wastewater discharge zones as major hotspots of SMC exposure and ecological risk. These findings highlight the importance of wastewater-derived SMCs as emerging contaminants in estuarine environments and demonstrate the utility of integrating long-term monitoring, explainable machine learning, and probabilistic risk assessment for ecological risk characterization.

## 1. Introduction

Synthetic musk compounds (SMCs) are a class of fragrance additives extensively used in personal care products, cosmetics, detergents, fabric softeners, and household cleaning products [1,2,3]. The widespread use of SMCs and their continuous release from consumer products have led to their ubiquitous distribution throughout the environment. SMCs have been detected in diverse environmental matrices, including surface waters, sediments, biota, ambient and indoor air, atmospheric particulate matter, and household dust [4,5,6]. Their occurrence in both outdoor and indoor environments indicates that human and ecological exposure may occur through multiple pathways, including inhalation, dermal contact, and dietary uptake [7]. The widespread atmospheric distribution of SMCs further suggests that environmental exposure is not limited to wastewater-impacted aquatic systems but may also extend to regions distant from primary emission sources [8]. Owing to their semi-volatile nature, certain SMCs can partition between air and water and undergo long-range environmental transport, leading to widespread environmental exposure beyond their original emission sources [9]. Their widespread consumption and continuous release through municipal wastewater systems have resulted in their ubiquitous occurrence in aquatic environments worldwide [10,11]. As a consequence, SMCs are increasingly recognized as emerging contaminants of environmental concern because of their persistence, bioaccumulation potential, and continuous discharge into receiving waters [12].

SMCs are generally classified into nitro musks, polycyclic musks, macrocyclic musks, and alicyclic musks. Among these groups [13], polycyclic musks such as HHCB and AHTN account for the majority of global consumption and are frequently detected in surface waters, sediments, wastewater effluents, and aquatic organisms [14,15]. Although the use of some nitro musks has been restricted in several countries due to concerns regarding toxicity and persistence [16], polycyclic musks remain widely used and continue to enter aquatic ecosystems through wastewater discharges [17,18,19].

The environmental behavior of SMCs raises particular toxicological concerns [20]. Their hydrophobic nature and relatively high octanol–water partition coefficients facilitate accumulation in sediments and biota, while resistance to biodegradation promotes long-term environmental persistence [21]. Numerous studies have reported bioaccumulation of SMCs in fish, shellfish, marine mammals, and human tissues [22,23,24,25]. Experimental studies have further demonstrated adverse effects including endocrine disruption, developmental toxicity, phototoxicity, and [26,27,28]. These characteristics suggest that long-term ecological risks may extend beyond those predicted from dissolved water concentrations alone [29].

In Korea, several studies have documented the occurrence of SMCs in rivers, wastewater treatment plants, sediments, aquatic organisms, and human biomonitoring samples [30,31,32,33,34]. Elevated concentrations of HHCB and other synthetic musks have been reported in the Han River watershed and adjacent coastal environments, indicating wastewater effluent as a major contamination source [35]. Nevertheless, information regarding long-term exposure patterns and ecological risks in estuarine ecosystems remains limited. Most previous investigations have focused on occurrence and distribution, whereas studies integrating environmental monitoring with quantitative ecological risk assessment remain scarce. The Han River estuary represents an environmentally important transitional zone that receives substantial urban wastewater inputs from the Seoul metropolitan area. Understanding the occurrence and ecological implications of SMC contamination in this system is therefore essential for evaluating potential environmental risks associated with wastewater-derived emerging contaminants. In addition, identifying environmental factors associated with SMC occurrence may improve monitoring efficiency and contribute to the development of risk-based surveillance strategies.

Therefore, the objectives of this study were to (1) characterize the spatial and temporal occurrence of twelve SMCs in the Han River estuary and adjacent coastal waters using a three-year monitoring dataset (2020–2022), (2) identify environmental factors influencing SMC distribution using XGBoost coupled with SHapley Additive exPlanations (SHAP) analysis, and (3) evaluate ecological risks through Monte Carlo-based probabilistic hazard quotient assessment. The findings provide new insights into the environmental exposure and ecological risks of synthetic musk compounds in estuarine environments and contribute to the risk characterization of wastewater-derived emerging contaminants.

## 2. Materials and Methods

### 2.1. Chemicals and Reagents

Standard reference materials for synthetic musk analysis were obtained from multiple commercial suppliers. NMs and PCMs, including musk ketone (MK), musk xylene (MX), musk ambrette (MA), musk moskene (MM), musk tibetene (MT), HHCB, and AHTN, were sourced from Sigma-Aldrich (St. Louis, MO, USA). Additional synthetic musk standards Phantolide (ABDI), Tonalid (AHDI), Cashmeran (DPMI), and Traesolide (ATII) were acquired from Toronto Research Chemicals (Toronto, ON, Canada). Iso E Super (OTNE) was procured from Supelco Inc. (Bellefonte, PA, USA). Comprehensive physicochemical properties of target analytes, encompassing chemical nomenclature, CAS registry numbers, molecular formulas, molecular weights, henry’s law constant, toxicity value, and structural configurations, are presented in Table S1. Deuterated fluoranthene (98 atom% D, Sigma-Aldrich) served as the internal standard for quantitative analysis. All organic solvents employed were high-performance liquid chromatography (HPLC) grade and obtained from Fisher Scientific (Waltham, MA, USA).

### 2.2. Sampling and Analytical Methods

**Figure 1:**
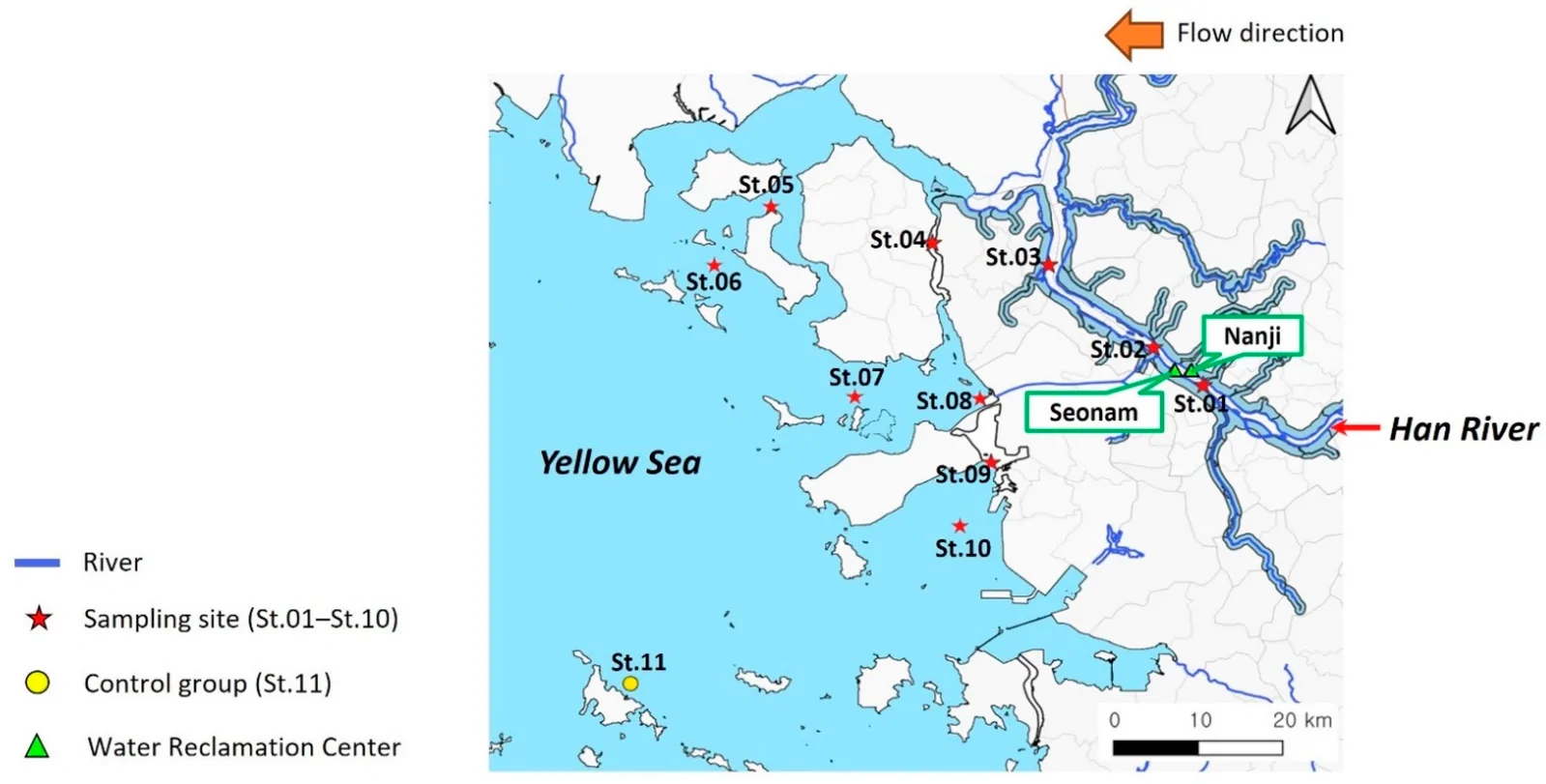
Geographic classification of monitoring stations: (1) St. 01–St. 03 located in the Han River outflow area, (2) St. 04–St. 06 positioned along Ganghwa Island coastline, (3) St. 07–St. 10 distributed across Incheon coastal waters, (4) St. 11 situated at Deokjeok Island. The map shows administrative boundaries, hydrographic features, coastlines, and sampling sites. It was created using QGIS (v. 3.16) with spatial data obtained from the V-World Spatial Information Open Platform [36].

The water column was selected as the target matrix in accordance with the temporal and risk assessment objectives of this study. A total of 222 water samples were collected from 13 designated monitoring stations (Figure 1, Table S2) during May, August, and October sampling campaigns conducted in 2021 and 2022. The monitoring network included three downstream Han River locations and nine coastal sites along the western shoreline. Two prominent water reclamation centers, Nanji (37.59° N, 126.85° E) and Seonam (37.58° N, 126.83° E), were strategically positioned between monitoring stations St. 01 and St. 02. Complete spatial distribution and coordinates of sampling locations are depicted in Figure 1. Freshwater influence in the Gangwha and Incheon coastal zones originates from the Yeomha and Seokmo channel systems, with additional impact from Han River outflow. Water depth was measured prior to sampling using a portable echo sounder (Hondex PS-7FL, Hondex Co., Ltd., Saitama, Japan). Water samples were subsequently collected from the surface and bottom water layers using a 5 L Niskin bottle sampler (General Oceanics, Miami, FL, USA). Surface collections targeted the upper 0.5 m water layer, while benthic samples originated from depths approximately 1 m above the substrate. Sample handling protocols involved immediate placement into 1 L amber glass containers with complete volume fill to eliminate air space, subsequently maintained at −2 °C pending laboratory analysis. A comprehensive sampling along the South Korean coast was undertaken with the Korea Institute of Coastal Ecology (KICE) within the framework of the Incheon Metropolitan Government-supported project, “Basic Research on the Han River Estuary Environment.”

Following the analytical methodology briefly described by Jo et al. [37], samples were extracted by liquid–liquid extraction and concentrated under a gentle stream of nitrogen using a nitrogen concentrator (Goojung EnT Co., Seoul, Republic of Korea). Instrumental analysis was conducted using an Agilent 7890B gas chromatograph coupled with a 5977A mass selective detector (Agilent Technologies, Santa Clara, CA, USA). The analysis was conducted using the shared research equipment program of the Korea Basic Science Institute (KBSI). Compound separation was accomplished through a DB-5 MS UI capillary column (5% diphenyl-95% dimethyl siloxane stationary phase, 30 m × 0.25 mm internal diameter, 0.25 µm film thickness) supplied by J&W Scientific (Santa Clara, CA, USA). Injection parameters included an inlet temperature of 280 °C with 1 µL sample volume introduced via splitless injection mode. Ultra-high purity helium (99.999%) served as the carrier gas at a constant flow rate of 1 mL/min. GC oven temperature program started at 60 °C, increased to 200 °C at 20 °C/min, then to 220 °C at 2 °C/min, where it was held for 10 min, and finally increased to 320 °C at 20 °C/min. The total analysis time was 32 min. Mass spectrometric detection employed electron ionization at 70 eV with transfer line and ion source temperatures maintained at 250 °C and 230 °C, respectively. Initial compound identification utilized full scan mode (m/z 40–600) for retention time and spectral confirmation, followed by quantitative determination using selected ion monitoring (SIM) mode with target and qualifier ions. Quality control and quality assurance (QA/QC) data is shown in Jo et al. [37]. Method detection limits (MDL) and method quantification limits (MQL) were determined from seven replicate analyses of spiked organic-free water, with MDL and MQL calculated as 3.14 and 10 times the standard deviation of the replicates, respectively [37]. The MDL ranged from 0.001 to 0.021 μg/L and the MQL from 0.003 to 0.066 μg/L across the twelve compounds; for HHCB, AHTN, and MK the MDL values were 0.012, 0.004, and 0.003 μg/L. Recoveries ranged from 74 to 115% with relative standard deviations of 2 to 18% [37]. Concentrations below the MDL were reported as not detected.

Water quality parameters including transparency, temperature, salinity, pH, dissolved oxygen (DO), chemical oxygen demand (COD), total organic carbon (TOC), suspended solids (SS), chlorophyll-a (Chl.a), nitrogen species (NH_4_-N, NO_2_-N, NO_3_-N, dissolved inorganic nitrogen (DIN)), total nitrogen (TN), phosphate (PO_4_-P), and total phosphorus (TP) were simultaneously measured with SMCs during the sampling period as shown in Table S3. All water quality parameters were determined in accordance with the Korean Standard Methods for Marine Environment [38]. Temperature, salinity, pH, and dissolved oxygen were measured in situ using a conductivity–temperature–depth (CTD) profiler (Ocean Seven 305, Idronaut S.r.l., Brugherio, Italy). The remaining parameters were determined in the laboratory using a UV–Vis spectrophotometer (UV-1800, Shimadzu Corporation, Kyoto, Japan) and an elemental analyzer (FLASH 2000, Thermo Fisher Scientific, Waltham, MA, USA).

### 2.3. Predictive Modeling, Risk Assessment, and Statistical Analysis

To examine the relationships between environmental parameters and SMC concentrations, an XGBoost regression model coupled with SHapley Additive exPlanations (SHAP) analysis was employed. XGBoost is a gradient boosting ensemble algorithm suited to datasets in which predictors are correlated and relationships are nonlinear [39], and SHAP is an interpretation framework based on cooperative game theory that decomposes each prediction into additive contributions from individual predictors [40]. A tree-based approach was adopted because the water quality parameters covary along the freshwater–seawater mixing gradient, so that linear coefficients cannot be attributed to individual variables, and because the relationships proved to be threshold-dependent rather than proportional. Gradient boosting was preferred to other ensemble methods because its regularized objective allows model complexity to be constrained explicitly, which is necessary at the present sample size, and because exact SHAP values can be computed for tree ensembles. This approach was adopted to rank the influence of routinely monitored water quality parameters rather than to predict SMC concentrations.

The XGBoost model was trained using seven environmental parameters as input features: DO, TOC, COD, SS, Chl.a, TN, and TP. These variables were chosen for their expected relationships with SMCs distribution and to maintain optimal model performance by avoiding overfitting from excessive variable inclusion. SMC concentrations were log-transformed using log(1 + x) to handle zero values and normalize the distribution. The dataset was split into training (80%) and testing (20%) sets with random stratification. Missing values in the environmental parameters were imputed using mean substitution prior to model training. The XGBoost model was configured with 100 estimators, maximum depth of 3, learning rate of 0.05, minimum child weight of 5, L1 regularization (α = 0.1), L2 regularization (λ = 1.0), subsample ratio of 0.8, and column sampling ratio of 0.8. These regularization parameters were introduced to prevent overfitting given the relatively modest sample size (*n* = 186), and model stability was confirmed through 5-fold cross-validation. Model performance was evaluated using R^2^ score, root mean square error (RMSE), and mean absolute error (MAE) on the test dataset. SHAP values were calculated for all test samples to quantify each parameter’s contribution to individual predictions and identify the most influential environmental factors affecting SMC concentrations. All analyses were conducted in Python 3.12.4 (Python Software Foundation, Wilmington, DE, USA) using the xgboost (v3.0.2), shap (v0.48.0), scikit-learn (v1.4.2), pandas (v2.2.2), numpy (v1.26.4), and matplotlib (v3.8.4) libraries. The model was trained solely on measured values obtained at the same station and depth (*n* = 148 for training and *n* = 38 for testing).

To assess potential ecological risks associated with synthetic musk contamination, a Monte Carlo simulation approach was employed to calculate hazard quotients (HQ) for three dominant compounds: HHCB, AHTN, and MK. Predicted No-Effect Concentrations (PNEC) values were derived from available No-Observed-Effect Concentrations (NOEC) using uncertainty factors (UF) according to Equation (1):

PNEC = NOEC/UF(1)

where NOEC is the no-observed-effect concentration (ng/L) obtained from chronic toxicity studies and UF is the assessment factor applied to account for interspecies variability and laboratory-to-field extrapolation. NOEC values were taken from the compilation of Guo et al. [41], who tabulated chronic endpoints for algae, cladocerans, and fish and selected the lowest value for each compound; in each case this was the fish endpoint. The values applied were HHCB (NOEC = 68,000 ng/L for Pimephales promelas [42,43], UF = 10, PNEC = 6800 ng/L), AHTN (NOEC = 43,458 ng/L for Pimephales promelas, as compiled by [40], UF = 10, PNEC = 4345.8 ng/L), and MK (NOEC = 32,304 ng/L for *Danio rerio* [44], UF = 50, PNEC = 646.08 ng/L). For comparison, PNEC values reported in the NORMAN Ecotoxicology Database [45] and HC5 values derived from species sensitivity distributions [20] were also compiled, and the sensitivity of the risk characterization to the choice of PNEC is examined in Section 3.3.

To incorporate analytical uncertainty into the risk assessment, a Monte Carlo simulation approach was employed. Measured environmental concentrations (MEC) at each sampling location and depth were treated as the central tendency of a lognormal distribution. The lognormal distribution parameters were defined as follows:

μ = ln(MEC)(2)

σ_log = √{ln(1 + RSD^2^)} (3)

where μ is the location parameter (i.e., the natural logarithm of the measured concentration), σ_log is the scale parameter (i.e., the log-standard deviation) derived from the relative standard deviation (RSD) of the analytical method, and MEC is the measured environmental concentration (μg/L) at a given station and depth. RSD values were obtained from QA/QC data: 0.10 for HHCB, 0.15 for AHTN, and 0.13 for MK. For each sampling location, depth, and compound, 10,000 random concentration values (C_sim) were generated from the lognormal distribution:

C_sim ~ Lognormal(μ, σ_log)(4)

Hazard quotients were then calculated for each simulated concentration:

HQ = C_sim/PNEC(5)

Statistical outputs from the 10,000 iterations included mean HQ, 95th percentile HQ, maximum HQ, and the probability of exceeding unity (P(HQ > 1)). All simulations were performed in Python 3.12.4 using the numpy and pandas libraries.

Differences among site groups were tested with the Kruskal–Wallis test followed by Dunn’s post hoc test with Bonferroni adjustment, differences between surface and bottom water with the Wilcoxon signed-rank test on paired samples, and interannual differences with the Kruskal–Wallis test. Nonparametric methods were adopted because the concentration data are strongly right-skewed and contain non-detects, which were treated as zero. Since samples were collected repeatedly at the same stations, the observations are not strictly independent, and the group comparisons are accordingly presented as descriptive rather than as inference from a fully specified error structure. Analyses were performed in Python 3.12.4 using the scipy and scikit-posthocs libraries, with significance assessed at α = 0.05.

## 3. Results

### 3.1. Spatial and Temporal Concentration Pattern

**Figure 2:**
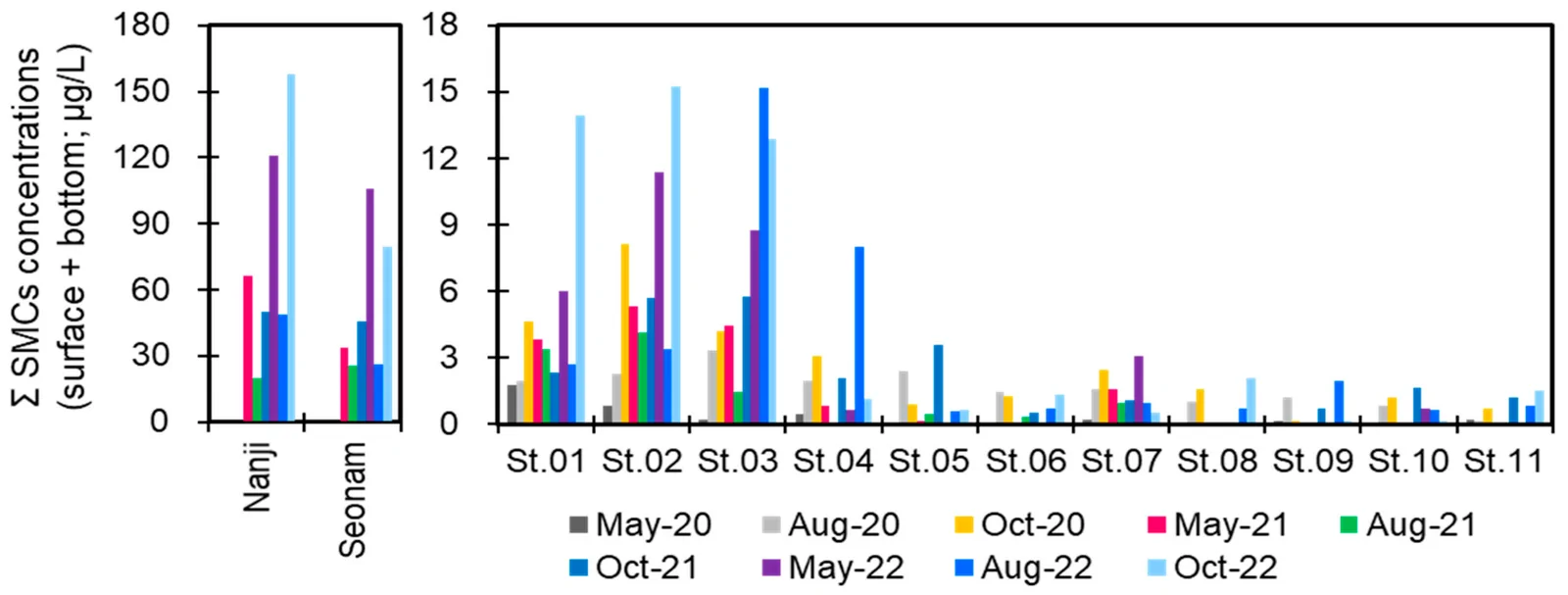
Temporal and spatial distribution of Σ12 SCM concentrations (μg/L) at Nanji and Senam Water Reclamation Centers and monitoring sites St. 01 through St. 11 from 2020 to 2022. Data for 2020 are referenced from Jo et al. [37].

The spatial and temporal distribution of Σ12 SMCs in the Han River estuary and adjacent coastal waters exhibited pronounced variability across sampling locations and seasons from 2021 to 2022 (Figure 2, Table 1). Combined concentrations of SMCs ranged from non-detectable levels to 157.6 μg/L, representing substantially elevated contamination compared to the 2020 baseline study (0.38–4.81 μg/L across all sampling sites; Jo et al. [37], particularly at point source locations. Surface and bottom concentrations did not differ significantly, either overall (Wilcoxon signed-rank test, *n* = 111, *p* = 0.749) or within any individual site group (*p* = 0.201–0.679); vertical distribution patterns for each station are provided in Text S1 and Table S3.

The highest concentrations were consistently observed at the two water reclamation centers, where Nanji demonstrated extreme temporal variation from 19.6 μg/L (August 2021) to 157.6 μg/L (October 2022) (Figure 2). Seonam showed similar dramatic increases, ranging from 25.5 μg/L (August 2021) to 106.1 μg/L (May 2022) (Figure 2). Several factors could account for this elevation, including increased consumption of synthetic musk-containing products, reduced removal efficiency, or changes in influent composition or discharge volume. These could not be distinguished here, since samples were collected from the effluent only and the operational records of the two facilities were not accessible. That the increase was confined to the discharge points and was not observed across the monitoring stations suggests a change within the facilities rather than a general rise in loading across the watershed, although paired influent and effluent sampling with concurrent discharge volume records would be required to establish this. Han River downstream stations also showed elevated concentrations compared to 2020, with combined levels reaching up to 15.3 μg/L in 2022 (Figure 2). In contrast, coastal monitoring sites (St. 04–St. 11) generally remained below 5 μg/L, similar to the 2020 study, and the distinct concentration gradient from point sources to coastal waters demonstrates the dominant influence of wastewater discharge on SMC distribution patterns. Concentrations differed significantly among the site groups (Kruskal–Wallis test, H = 128.6, *p* < 0.001). Dunn’s post hoc test distinguished three tiers: the water reclamation centres (median 25.69 μg/L) from the Han River downstream stations (1.88 μg/L, *p* = 0.013), and both from the coastal groups (0.07–0.34 μg/L, *p* < 0.001), whereas the Ganghwa, Incheon, and Deokjeok groups did not differ from one another (*p* = 1.000). This grouping corresponds to the management priority zones delineated in Section 3.3.

Temporal concentration trends revealed distinct patterns over the three-year monitoring period (Figure S1). The 2020 baseline [37] showed relatively low concentrations with consistent seasonal patterns, where October concentrations (2.58 ± 2.23 μg/L) were notably higher than May (0.37 ± 0.50 μg/L) and August (1.65 ± 0.84 μg/L). In 2021, seasonal variation was less pronounced, with relatively similar levels across May (1.49 ± 1.96 μg/L), August (1.00 ± 1.39 μg/L), and October (2.24 ± 1.90 μg/L). Mean concentrations were higher in 2022 across all seasons, with May (2.80 ± 3.89 μg/L), August (3.24 ± 4.31 μg/L), and October (4.51 ± 5.87 μg/L) exceeding those of previous years. This increase was statistically significant at the water reclamation centres (Kruskal–Wallis test, *p* = 0.005), where median concentrations rose from 18.47 μg/L in 2021 to 49.38 μg/L in 2022, but not across the monitoring stations (*p* = 0.230), indicating that the trend is driven primarily by the treatment facilities. Mean concentrations in May 2022 were comparable to the October values of earlier years, suggesting a weakening of the seasonal pattern, although the interannual differences at the monitoring stations were not statistically significant and this observation should be regarded as tentative. Interannual variation in precipitation and freshwater discharge would be expected to influence the seasonal contrast through dilution, as reported previously for this system [37], but concurrent hydrological records were not compiled here and the contribution of these factors could not be evaluated.

From a management perspective, the shift toward more consistently elevated concentrations suggests that SMC loading to the estuary may be increasing and that existing monitoring protocols—which typically do not include SMCs—are not capturing this trend. In Korea, routine surveillance of the study area falls under two national programs: the Water Environment Monitoring Network, operated by the National Institute of Environmental Research for riverine and estuarine waters, and the Marine Environment Monitoring Network, operated by the Korea Marine Environment Management Corporation for coastal waters. Both measure conventional parameters including nutrients, organic matter, and heavy metals, but neither includes SMCs or other fragrance-related emerging contaminants. The three-year upward trend documented in this study, particularly the substantial concentration increases at wastewater treatment facilities (e.g., Nanji: 19.6 μg/L in August 2021 to 157.6 μg/L in October 2022), supports the consideration of incorporating SMCs into routine water quality surveillance at sites receiving significant wastewater inputs. Furthermore, the weakening of seasonal concentration patterns observed in 2022, where May concentrations reached levels previously seen only in October, suggests that monitoring strategies based on seasonal sampling campaigns may not adequately capture year-round contamination dynamics. Transitioning from seasonal to more frequent or continuous monitoring at key discharge-influenced stations would improve the ability to detect emerging contamination trends. The substantially higher concentrations at wastewater treatment facilities compared to downstream and coastal stations point to wastewater effluent as a key area for management attention, though the extent to which current treatment processes remove SMCs was not assessed in this study. The spatial concentration gradient, combined with the detection of elevated MK risk levels at the remote St. 11 site (discussed in Section 3.3), suggests that monitoring programs for SMCs should encompass both proximal discharge zones and distant coastal areas to adequately capture the full extent of contamination.

**Figure 3:**
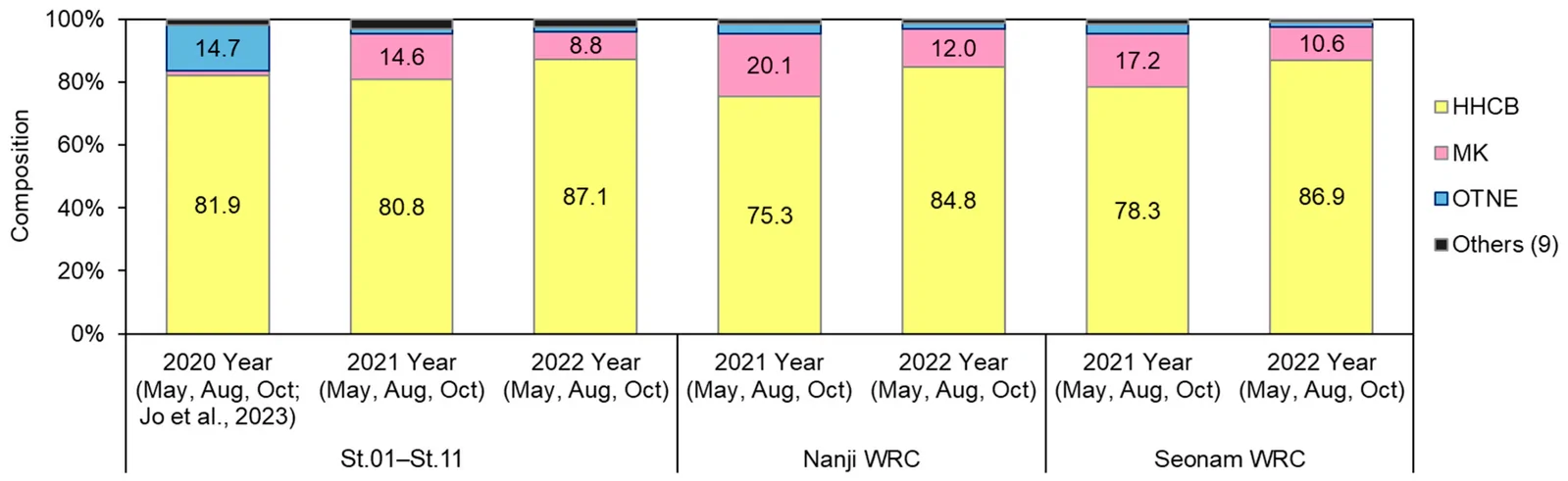
Compositional profiles of SMCs in the Han River estuary (surface + bottom) by region and sampling year (2020–2022). Stacked bar chart shows relative contributions of major compounds: HHCB (yellow), MK (pink), OTNE (blue), and Others comprising 9 minor compounds (gray). Data for 2020 from Jo et al. [37].

The compositional analysis revealed a consistent dominance of HHCB across all sampling periods and locations, accounting for 75.3–87.1% of total SMC concentrations (Figure 3). Notable compositional shifts occurred between 2020 and subsequent years: in 2020, OTNE was the secondary component representing 14.7% of total concentrations, while MK had minimal contribution (1.6%). From 2021 onwards, MK emerged as the second most abundant compound with substantial spatial and temporal variations ranging from 8.8% to 20.1%, while OTNE contributions decreased to 1.5–3.1%. The remaining nine compounds collectively represented 1.3–3.1% of total concentrations.

HHCB and MK concentrations in Han River downstream waters were higher than most previously reported values from both Korean and international waterways; detailed compound-by-compound comparisons are provided in Text S2 and Table 1. SMC concentrations near wastewater treatment facilities were comparable to or higher than those reported in sewage-impacted European rivers, although direct comparison is limited by differences in sampling locations and analytical methods. Notably, neither Korean nor European water quality standards currently include SMCs as regulated parameters; existing EU restrictions on certain nitro musks apply to cosmetic products rather than aquatic environments. This shared regulatory gap underscores the broader need for management frameworks that can translate growing monitoring evidence into actionable water quality criteria.

### 3.2. Identification of Surrogate Indicators for SMC Management Using XGBoost-SHAP Analysis

The correlation analysis between water quality variables and SMC concentrations revealed that nitrogen-related parameters exhibited the strongest positive correlations with SMCs (Table S4). These associations are interpreted throughout as indicators of shared wastewater loading rather than as evidence of mechanistic linkage, for the reasons set out below. TN showed consistently high correlation coefficients across sampling periods (r = 0.66–0.95), while DIN demonstrated similar patterns (r = 0.60–0.93). Salinity consistently showed strong negative correlations (r = −0.33 to −0.90), reflecting the dilution effect of seawater intrusion. The remaining parameters showed variable correlation patterns depending on sampling period and depth; detailed results are provided in Table S4.

However, in an estuarine system receiving wastewater discharge, multiple parameters co-vary along the freshwater–seawater mixing gradient, and these correlations may largely reflect conservative dilution of a common wastewater plume rather than mechanistic linkages. The correlation analysis therefore serves as an exploratory step to identify candidate variables for subsequent machine learning analysis rather than as evidence of any process linking the parameters to SMC fate. To capture nonlinear relationships and interaction effects beyond the limitations of linear correlation, XGBoost-SHAP analysis was performed using the combined dataset (*n* = 186) comprising 2021–2022 monitoring data and 2020 baseline data from Jo et al. [37], with model configuration as described in Section 2.3. Before these relationships are interpreted, it should be emphasized that TN and SMCs share a common origin in municipal wastewater and therefore co-vary with the wastewater fraction of the water mass. The influence of TN identified by the model reflects this shared dependence and not any process by which nitrogen affects SMC fate.

**Figure 4:**
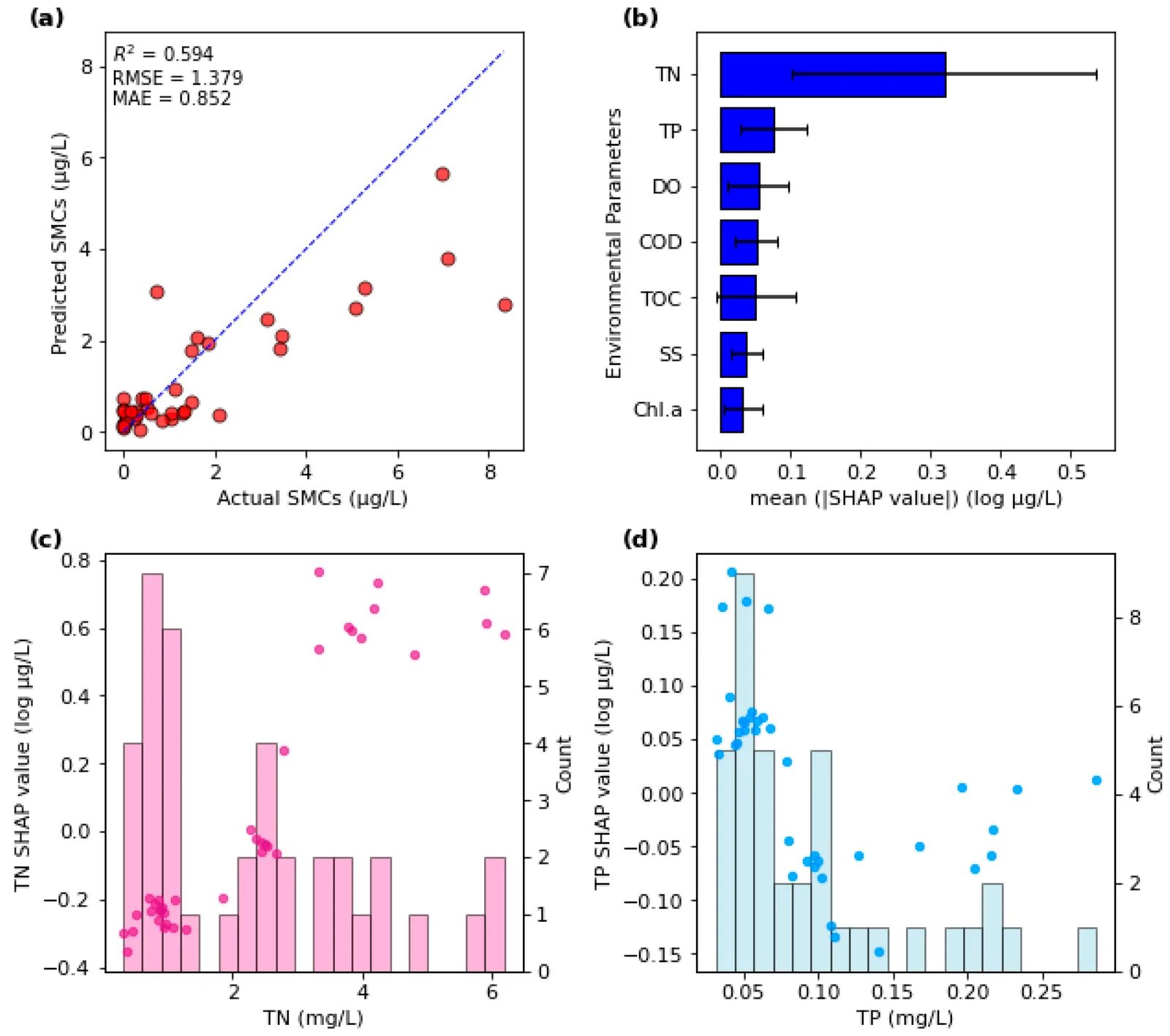
XGBoost model performance and SHAP analysis for SMCs concentration prediction: (**a**) predicted versus actual SMC concentrations, where the blue dashed line denotes the 1:1 line of perfect agreement; (**b**) feature importance ranking based on mean absolute SHAP values, with error bars indicating one standard deviation; (**c**) SHAP value distribution for TN; and (**d**) SHAP value distribution for TP. In (**c**,**d**), the points represent the SHAP value of each sample (left axis) and the bars represent the frequency distribution of the corresponding parameter (right axis); colours are used only to distinguish the two parameters and carry no additional meaning.

The model demonstrated reasonable predictive performance, with R^2^ = 0.59, RMSE = 1.38, and MAE = 0.85 on the held-out test set after back-transformation to the original concentration scale, and a 5-fold cross-validated R^2^ of 0.49 ± 0.05 on the log-transformed response used for fitting (Figure 4a, Table S5). The two values are computed on different scales and are therefore not directly comparable. The moderate R^2^ reflects the inherent complexity of predicting SMC concentrations from conventional water quality parameters alone, as SMCs are influenced by additional factors not captured in routine monitoring, including product usage patterns and wastewater treatment variability. Nevertheless, the primary objective of the modeling exercise was not to achieve precise concentration predictions but to identify the relative importance of environmental variables as potential surrogate indicators through SHAP analysis. Model performance was evaluated on a test set withheld from training and through 5-fold cross-validation, and residual analysis revealed no systematic bias, indicating that the fitted relationships are not attributable to overfitting. Validation was internal, however, as no independent dataset providing co-located SMC and water quality measurements was available for this region, and the transferability of the variable rankings to other estuarine systems requires further verification.

SHAP analysis identified TN as the most influential parameter (mean |SHAP| = 3.19 × 10^−1^, feature importance = 3.57 × 10^−1^), followed by TP (mean |SHAP| = 7.45 × 10^−2^, feature importance = 1.31 × 10^−1^) (Figure 4b, Table S5). The remaining five variables showed considerably lower and comparable contributions (mean |SHAP| = 3.12–5.29 × 10^−2^; Table S5). TN exhibited concentration-dependent behavior, with negative SHAP values at lower concentrations (<2 mg/L) transitioning to consistently positive contributions at higher concentrations (Figure 4c), suggesting threshold-type relationships driven by competing dilution and co-transport processes. TP showed a similar but weaker pattern, with both positive and negative contributions depending on concentration levels (Figure 4d). These concentration-dependent relationships likely reflect the influence of multiple competing processes on SMC distribution, including dilution effects at lower nutrient concentrations and co-transport with nutrient-rich wastewater at higher concentrations.

The dominance of TN is supported by a mechanistic rationale grounded in shared source characteristics. Municipal wastewater simultaneously carries nitrogen compounds from human metabolic waste and SMCs from personal care products such as shampoos, detergents, and fabric softeners [1,3]. Because both substance classes enter the sewer system through the same domestic pathway, their concentrations in receiving waters co-vary in proportion to the wastewater fraction of the water mass. This co-discharge mechanism is consistent with the spatial patterns observed in this study: stations receiving direct wastewater influence (St. 01–St. 03) showed simultaneously high TN (3.71–4.83 mg/L) and elevated SMC concentrations (2.24–5.03 μg/L), whereas distant coastal stations (St. 06–St. 11) exhibited low TN (0.29–0.85 mg/L) and correspondingly low SMC levels (0.07–0.76 μg/L) (Table S3). Furthermore, conventional activated sludge processes achieve only partial removal of both polycyclic musks and nitrogen, with substantial fractions passing through to effluent [14,46], indicating that TN and SMCs share not only the same point of entry but also similar behavior during wastewater treatment.

It is important to emphasize that TN is not proposed as a direct predictor of individual SMCs. No biogeochemical pathway links nitrogen cycling to SMC fate; the statistical association reflects their shared dependence on wastewater loading as a common external driver. The practical value of TN lies in serving as a readily available indicator of wastewater influence intensity, enabling environmental managers to flag stations warranting targeted SMC analysis using routinely collected monitoring data without additional cost. This approach is particularly relevant given that dedicated SMC monitoring programs have not yet been established in most countries, including Korea.

However, this surrogate relationship is expected to hold primarily in systems where municipal wastewater discharge is the dominant nitrogen source. In watersheds with significant agricultural runoff or industrial nitrogen inputs, elevated TN concentrations may not correspond to elevated SMC levels. Therefore, the applicability of TN as a preliminary screening indicator should be verified on a site-specific basis before adoption in other systems. Direct SMC measurement remains essential for confirmatory assessment, and future studies should evaluate this relationship across broader hydrological conditions and watershed land-use types.

### 3.3. Risk-Based Management Prioritization

To establish risk-based management priorities for synthetic musk contamination in the Han River estuary, Monte Carlo-based probabilistic risk assessment was conducted, revealing significant spatial variations in hazard quotients across the study area with distinct risk patterns for different synthetic musk compounds. Risk levels were categorized as: No Risk (HQ < 0.01), Probable Low Risk (0.01 ≤ HQ < 0.1), Expected Medium Risk (0.1 ≤ HQ < 1.0), and High Risk (HQ ≥ 1.0). The method detection limits for HHCB, AHTN, and MK lie two to three orders of magnitude below the corresponding PNEC values and below the concentrations equivalent to a hazard quotient of 0.01, so that samples reported as not detected cannot correspond to concentrations of ecological concern.

The quantitative assessment revealed that wastewater treatment facilities pose severe ecological risks, particularly for HHCB and MK. HHCB showed mean HQ values of 4.41 (surface) and 4.95 (bottom) at Nanji, and 4.32 (surface) and 3.33 (bottom) at Seonam. MK demonstrated even higher risks with mean HQ values of 7.79 (surface) and 9.55 (bottom) at Nanji, and 5.41 (surface) and 5.07 (bottom) at Seonam. In contrast, AHTN presented minimal risks with maximum mean HQ values of 0.07 (surface) at Nanji and 0.05 (surface) at Seonam. Among monitoring stations, Han River downstream sites (St. 01–St. 03) showed medium risk for HHCB (mean HQ = 0.34–0.44 for surface; 0.25–0.50 for bottom) and MK (mean HQ = 0.27–0.54 for surface; 0.27–0.57 for bottom), while coastal stations (St. 04–St. 11) demonstrated low risks below 0.20 for all compounds, with the exception of MK in surface water at St. 06 (mean HQ = 0.50) and in bottom water at St. 11. Notably, St. 11 (bottom) was the only monitoring station approaching the high risk threshold for MK with mean HQ = 0.70 and maximum HQ reaching 1.07.

Coastal stations (St. 04–St. 11) generally exhibited low risk levels for all compounds, with the notable exception of St. 11, where bottom-water MK showed a mean HQ of 0.70 approaching the high-risk threshold. This unexpected finding at the most distant monitoring site suggests that SMC contamination and associated risks may extend further into coastal waters than proximity to discharge points alone would predict, reinforcing the need for monitoring coverage beyond the immediate vicinity of treatment facilities.

**Figure 5:**
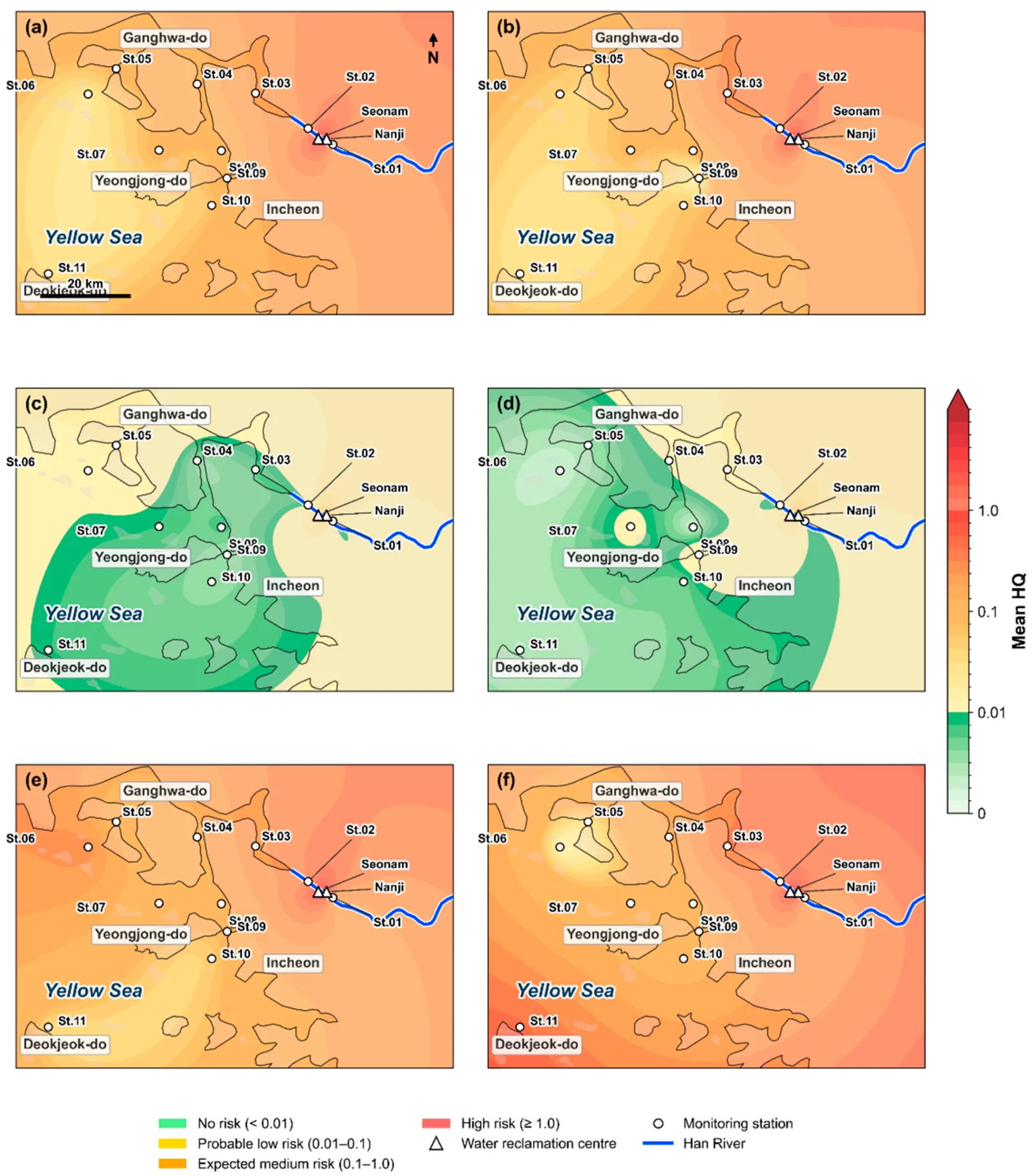
Spatial distribution of hazard quotients (HQ) for synthetic musk compounds in the Han River estuary. (**a**) HHCB surface waters, (**b**) HHCB bottom waters, (**c**) AHTN surface waters, (**d**) AHTN bottom waters, (**e**) MK surface waters, and (**f**) MK bottom waters. Monitoring stations are shown as circles and the Nanji and Seonam water reclamation centres as triangles. Risk levels are categorized as No Risk (green, HQ < 0.01), Probable Low Risk (yellow, 0.01 ≤ HQ < 0.1), Expected Medium Risk (orange, 0.1 ≤ HQ < 1.0), and High Risk (red, HQ ≥ 1.0). The Han River is indicated in blue. The scale bar and north arrow in panel (**a**) apply to all panels.

Spatial interpolation using Radial Basis Function (RBF) with linear function was employed to generate continuous risk distribution maps from discrete sampling points, creating 300 × 300 grids with values clipped to a maximum of 1.0 to prevent unrealistic extrapolation. Figure 5 illustrates the spatial distribution of hazard quotients for HHCB, AHTN, and MK across surface and bottom waters, clearly demonstrating distinct risk hotspots centered around the Nanji and Seonam wastewater treatment facilities. HHCB shows pronounced risk gradients in both surface (Figure 5a) and bottom waters (Figure 5b), with high-risk zones (red areas) at treatment facilities transitioning to medium-risk zones (orange areas) along Han River downstream stations and low-risk levels (green areas) at coastal sites. AHTN displays consistently low risk levels across the entire study area for both surface (Figure 5c) and bottom waters (Figure 5d), with predominantly green coloration. MK exhibits the most dramatic risk contrasts, showing extreme high-risk zones at treatment facilities for both surface (Figure 5e) and bottom waters (Figure 5f), with intense red coloration at Nanji and Seonam.

The spatial risk assessment confirms that synthetic musk contamination follows a clear dilution gradient from wastewater point sources to coastal waters, with potential ecological impacts concentrated near treatment facilities while coastal monitoring sites remain within acceptable risk ranges. The pronounced risk hotspots at Nanji and Seonam facilities indicate the critical need for enhanced synthetic musk removal technologies at wastewater treatment plants to protect aquatic ecosystem health. While current contamination levels at offshore monitoring stations suggest limited immediate ecological threats to marine environments, the consistent detection of medium-risk zones along the Han River downstream areas warrants continued monitoring and source control measures to prevent further environmental degradation.

The water-phase HQ values should be interpreted considering the physicochemical properties of the target compounds. HHCB exhibits high lipophilicity (log Kow = 5.9), was classified as not readily biodegradable [52], and shows fish bioconcentration factors of 600–1600 L/kg [53], suggesting potential for sediment accumulation and trophic transfer not captured by ambient concentration-based risk assessment. AHTN shares similar characteristics (log Kow = 5.4–5.7; not readily biodegradable) and has been reported to be more toxic to aquatic organisms than HHCB [54]. MK, despite a lower log Kow (4.3), showed a BCF of 1380 L/kg in fish [55] and poses additional concerns due to phototoxicity and long-term inhibition of multidrug transporters [16]. Given these properties, a considerable fraction of the SMC burden in the estuary is likely to reside in bottom sediments and in biota, and the bioconcentration factors cited above indicate enrichment of two to three orders of magnitude relative to the dissolved phase. The water-phase HQ values reported here may therefore be more representative of pelagic than of benthic exposure. Sediment-based risk characterization was not attempted in this study, as it would require organic carbon–normalized sediment PNEC values, which have been derived for HHCB and AHTN elsewhere [29], together with measured sediment organic carbon contents that were not available here. Future assessments in this system would benefit from combined sediment and biota measurements together with multimedia fate modeling that accounts for partitioning, bioaccumulation, and degradation kinetics.

Several limitations of this risk characterization warrant explicit statement. The PNEC values applied here were derived from single chronic endpoints with assessment factors, an approach that provides a screening-level estimate but does not represent the range of sensitivity across taxa. Each value rests on a freshwater fish endpoint; no algal endpoint was available for MK, and no marine or estuarine species was represented, although the study area is a brackish system. To examine how far the conclusions depend on this choice, hazard quotients were recalculated using HC5 values derived from species sensitivity distributions for the same three compounds [20] (Table 2). When the HC5 obtained from the combined acute and chronic dataset was applied, the spatial pattern and the risk classification were unchanged, with hazard quotients exceeding unity only at the water reclamation centres. When the HC5 obtained from the chronic dataset alone was applied, the hazard quotients for MK increased by more than two orders of magnitude and exceeded unity at all stations. The chronic distribution for that compound differs by three orders of magnitude from the distribution fitted to the combined dataset, a divergence not observed for HHCB or AHTN, and this result is therefore more plausibly attributed to the limited number of chronic endpoints available for MK than to widespread ecological risk. The assessment-factor values were accordingly retained as the primary basis, and the distribution-based values are reported as a sensitivity analysis. A comparable comparison of alternative PNEC derivations has been applied to triclosan in sludge-amended soils, where values obtained from direct toxicity data, from equilibrium partitioning, and from published distributions were evaluated side by side [56]. Refinement of the present assessment would require chronic toxicity testing with estuarine species, characterization of the sediment-associated fraction, and consideration of joint toxicity, since the compounds were treated individually here whereas they occur as a mixture.

Based on the spatial distribution of the compound-specific hazard quotients described above, three management priority zones can be delineated for the Han River estuary system. Management Priority Zone 1 (Immediate Action) encompasses the Nanji and Seonam water reclamation centers, where mean HQ values for HHCB (3.33–4.95) and MK (5.07–9.57) consistently exceeded unity across both surface and bottom waters. These facilities represent the most critical intervention points, where evaluation of advanced treatment technologies for SMC removal and implementation of effluent-specific SMC monitoring should be prioritized. Management Priority Zone 2 (Enhanced Surveillance) includes the Han River downstream stations (St. 01–St. 03), where HHCB and MK showed medium-risk levels (mean HQ = 0.26–0.57) with HQ values approaching but generally not exceeding unity. These stations warrant regular SMC monitoring at increased frequency to detect potential risk escalation, particularly given the upward concentration trends documented in Section 3.1. Management Priority Zone 3 (Routine Monitoring) covers the coastal stations (St. 04–St. 10), where mean HQ remained below 0.20 for all compounds except MK in surface water at St. 06 (0.50), which nonetheless remained well below unity. Periodic surveillance at these stations is sufficient under current conditions, though the anomalous risk elevation at St. 11 (discussed below) indicates that even distant coastal sites should not be excluded from monitoring networks entirely.

The elevated MK risk levels observed at St. 11 (Deokjeok Island), despite its background coastal location approximately 50 km from the Han River outflow, suggests multiple contributing factors. The hydrodynamic conditions in the Yellow Sea may influence contaminant distribution patterns, though the specific transport mechanisms for synthetic musks require further investigation. Additionally, Deokjeok Island’s position along major shipping routes could expose it to vessel-related contamination, while atmospheric deposition or inputs from other regional sources cannot be excluded. These findings highlight the need for comprehensive source identification studies to better understand synthetic musk distribution patterns in coastal environments, as the contamination at this background site indicates that point source influence may extend beyond the immediate vicinity of major discharge locations.

From a management standpoint, this finding suggests that management strategies focused solely on proximal wastewater discharge points may be insufficient. Comprehensive SMC management in coastal environments should also consider long-range transport pathways and diffuse sources, potentially requiring coordination between local watershed management authorities and regional marine environmental management agencies.

The hazard quotients reported here were derived for each compound separately, and no combined risk was calculated. Since HHCB, AHTN, and MK co-occur at every station, these values characterize single-substance exposure rather than the mixture to which aquatic organisms are exposed, and they therefore understate the total burden, which also includes wastewater-derived contaminants not measured in this study. Concentration addition would provide a conventional first-tier estimate of combined exposure, but it presumes a common mode of action and non-interacting effects, neither of which has been demonstrated for these structurally distinct compounds. Greater-than-additive effects cannot be excluded on mechanistic grounds, since musks have been reported to inhibit multidrug transporters over prolonged exposure [16], a mechanism that could elevate the internal burden of co-occurring contaminants beyond what quotient-based approaches predict. Characterizing mixture effects in this system would require experimental work with defined combinations.

## 4. Conclusions

This study provides a comprehensive assessment of the occurrence, driving factors, and ecological risks of synthetic musk compounds (SMCs) in the Han River estuary through the integration of long-term monitoring, machine learning analysis, and probabilistic risk assessment. Among the target compounds, HHCB consistently dominated the SMC profile, while musk ketone (MK) emerged as the second most abundant compound during the later monitoring period. Increasing concentrations at wastewater treatment facilities and the attenuation of seasonal variability in 2022 indicate that SMC contamination is becoming more persistent and less dependent on seasonal hydrological conditions.

Machine learning analysis identified total nitrogen (TN) as the most influential predictor of SMC occurrence. Rather than reflecting a direct biogeochemical relationship, TN appears to function as a proxy indicator of municipal wastewater influence, highlighting the dominant role of wastewater effluents as a source of SMC contamination in the estuarine environment. Given that TN is routinely monitored in national water-quality programs, it may provide a practical screening tool for identifying areas vulnerable to SMC contamination, although further validation is required across watersheds with differing land-use characteristics and nitrogen sources.

Probabilistic ecological risk assessment revealed spatially heterogeneous risk patterns. Wastewater treatment facilities, particularly the Nanji and Seonam plants, were identified as high-priority management zones where hazard quotients for HHCB and MK frequently exceeded acceptable thresholds. In contrast, downstream estuarine and coastal sites generally exhibited lower risk levels, although localized risk elevation at St. 11 suggests that contamination hotspots may occur beyond the immediate vicinity of discharge points. These findings emphasize the importance of risk-based monitoring strategies that extend beyond conventional point-source assessments.

From a toxicological and environmental management perspective, the continued presence of HHCB and MK at concentrations associated with potential ecological effects raises concerns regarding chronic exposure of aquatic organisms in wastewater-influenced estuarine systems. The observed contamination patterns underscore the need for improved source control and enhanced removal of SMCs at wastewater treatment facilities.

Although the present study was limited to selected treatment facilities and did not explicitly evaluate contributions from tributaries, industrial sources, or other wastewater discharges within the Han River watershed, the integrated monitoring–modeling–risk assessment framework developed herein provides a practical approach for identifying contamination hotspots and prioritizing management actions. This framework may be broadly applicable to other urbanized estuarine environments where emerging contaminants are insufficiently monitored but pose potential ecological risks.

## Figures and Tables

**Table 1:** Summary of prior research on selected SMCs (HHCB, AHTN, MK, OTNE) concentrations in rivers and aquatic systems by country (μg/L).

Country	Location	Sampling Period	HHCB	AHTN	MK	OTNE	Reference
South Korea	Han River Downstream	2021 May–Oct	ND *–3.42	ND–0.11	ND–0.57	ND–0.07	(This study)
	Han River Downstream	2022 May–Oct	ND–7.30	ND–0.10	ND–1.19	ND–1.19	(This study)
	Han River Downstream	2020 May–Nov	ND-4.41	ND	ND–0.23	ND–0.46	[37]
	Tributary	2018 May–Nov	0.017–2.825	ND–0.169	0.005–0.258	NA **	[46]
	Han River		0.026–0.705	ND–0.046	ND	NA	
	Nakdong River	2009 Apr–Oct	0.11–1.06	0.10–0.17	NA	1.27–1.29	[47]
		2007 Oct, 2008 Apr	2.56–4352	0.55–1.21	NA	NA	[32]
Italy	Molgora River	2010 Nov	<0.05–1.14	<0.25–0.36	NA	NA	[48]
India	Kaveri	2015 Nov, 2016 May	0.001–0.200	0.001–0.077	0.006–0.020	NA	[49]
	Vellar		0.002–0.017	0.001–0.006	0.003–0.032	NA	
	Thamiraparani		0.006–0.020	0.003–0.032	0.003–0.062	NA	
China	Haihe River	2008 Dec.	0.035–0.032	0.023–0.027	NA	NA	[13]
Germany	River Ruhr	2006 Sep	0.016–0.960	0.003–0.250	0.006–0.300	0.029–0.810	[50]
Rumania	Somes River	-	0.172–0.313	0.81–0.11	NA	NA	[51]
China	LakeTaihu	2009 Jun–Sep	0.003–0.212	0.071–0.006	0.013–0.080	NA	[41]

* Not Detected; ** Not Analyzed.

**Table 2:** Comparison of PNEC values for HHCB, AHTN, and MK and the resulting mean hazard quotients.

	HHCB	AHTN	MK
PNEC (ng/L)			
Assessment factor (this study)	6800	4345.8	646.08
HC5, acute and chronic data [20]	8409	3154	1211
HC5, chronic data only [20]	1331	979	1.2
Mean HQ, water reclamation centres			
Assessment factor	3.33–4.95	0.05–0.08	5.07–9.57
HC5, acute and chronic	2.69–4.01	0.06–0.11	2.71–5.10
HC5, chronic only	17.00–25.33	0.20–0.35	2733–5137
Mean HQ, monitoring stations			
Assessment factor	0.01–0.50	0.002–0.030	0.02–0.70
HC5, acute and chronic	0.01–0.41	0.003–0.040	0.01–0.37
HC5, chronic only	0.07–2.57	0.01–0.11	8.16–374.70

Note: Hazard quotients were obtained by Monte Carlo simulation (10,000 iterations per station, layer, and compound) under each PNEC derivation. Values are the range of station-level mean HQ within each group. Mean HQ exceeded unity in 8 of 78 cases under the assessment-factor and combined HC5 derivations, and in 36 of 78 cases under the chronic-only HC5 derivation.

## Data Availability

The data presented in this study are available on request from the corresponding author.

## Supplementary Materials

The following supporting information can be downloaded at https://www.mdpi.com/article/10.3390/toxics14080718/s1, Text S1: Vertical distribution patterns of synthetic musk compounds across sampling stations in the Han River estuary; Text S2: Comparison of synthetic musk compound concentrations with previously reported values from Korean and international waterways [13,41,46,47,48,49,50]; Table S1: Chemical properties, molecular characteristics, and toxicological parameters of synthetic musk compounds analyzed in this study (sources: National Institute of Standards and Technology (NIST) Chemistry WebBook and CompTox Chemicals Dashboard v2.5.3, United States Environmental Protection Agency (US EPA)); Table S2: Sampling site information including coordinates, location descriptions, distances from wastewater treatment centers, and total sample numbers; Table S3: Mean values of selected water quality parameters and total concentrations of SMC by sampling station and depth layer (surface/bottom) during 2021–2022; Table S4: Pearson correlation coefficients between SMC concentrations and water quality parameters across different sampling periods (2021–2022) and depths (surface and bottom layers). Asterisks denote statistical significance (* *p* < 0.05, ** *p* < 0.01); Table S5: XGBoost-SHAP analysis results for environmental parameters affecting SMC concentrations; Figure S1: Temporal variation in Σ12 SMC concentrations in the Han River estuary and coastal waters from 2020 to 2022 (excluding water reclamation centers). Data for 2020 from Jo et al. [37].
