# Supplementary material for: Probabilistic Ecological Risk Assessment of Synthetic Musk Compounds in the Han River Estuary: Integrating Multi-Year Monitoring and Explainable Machine Learning
Source: Toxics. 2026 Aug 13;14(8):718. doi: 10.3390/toxics14080718 (PMC13517401; doi:10.3390/toxics14080718)
Supplement: Supplementary file 1 [file toxics-14-00718-s001.zip › toxics-4430795-supplementary.pdf]

Supplementary

Integrating multi-year monitoring, machine learning diagnostics, and probabilistic risk assessment to inform management of synthetic musk compounds in the Han River estuary

Text S1. Vertical distribution patterns of synthetic musk compounds across sampling stations in the Han River estuary

Vertical distribution analysis revealed that bottom water concentrations generally exceeded surface concentrations at most sampling sites, particularly at the water reclamation centers (Table S2). At Nanji, bottom water concentrations (26.60 μg/L) were comparable to surface concentrations (26.03 μg/L), while Seonam showed higher bottom concentrations (41.26 μg/L) compared to surface concentrations (35.95 μg/L). This pattern was also evident at Han River stations (St.01–St.03), where bottom water typically contained higher SMC concentrations than surface water, with St.01 showing bottom concentrations of 2.24 μg/L versus surface concentrations of 3.13 μg/L, St.02 demonstrating 3.90 μg/L (bottom) versus 3.63 μg/L (surface), and St.03 exhibiting 5.03 μg/L (bottom) compared to 3.06 μg/L (surface). However, coastal monitoring sites (St.04–St.11) showed more variable vertical distribution patterns, with some sites like St.05 showing higher surface concentrations (0.64 μg/L) than bottom concentrations (0.25 μg/L), while others like St.11 demonstrated higher bottom concentrations (0.41 μg/L) compared to surface concentrations (0.21 μg/L).

Text S2. Comparison of synthetic musk compound concentrations with previously reported values from Korean and international waterways

The SMC concentrations observed in this study showed variable patterns when compared to previous monitoring efforts (Table 1). HHCB concentrations in Han River downstream waters (ND–7.30 μg/L) were substantially higher than earlier Korean studies, including Han River tributary monitoring (0.017–2.825 μg/L) [46] and Nakdong River surveys (0.11–1.06 μg/L) [47], and also exceeded most international waterways, including Chinese studies (0.035–0.032 μg/L for Haihe River and 0.003–0.212 μg/L for Lake Taihu) [13, 41], Italian Molgora River (<0.05–1.14 μg/L) [48], German River Ruhr (0.016–0.960 μg/L) [50], and MK concentrations (ND–1.19 μg/L) substantially exceeded previous Korean Han River tributary measurements (0.005–0.258 μg/L) [46] and also surpassed international levels, including Indian rivers (0.003–0.062 μg/L) [49], German River Ruhr (0.006–0.300 μg/L) [50], and Chinese Lake Taihu (0.013–0.080 μg/L) [41]. AHTN levels (ND–0.11 μg/L) were comparable to previous Korean measurements (ND–0.169 μg/L) [46] and fell within ranges of international studies, including Indian rivers (0.001–0.077 μg/L) [49] and German River Ruhr (0.003–0.250 μg/L) [50]. OTNE concentrations (ND–1.19 μg/L) were comparable to earlier Korean Nakdong River studies (1.27–1.29 μg/L) [47] but exceeded German River Ruhr levels (0.029–0.810 μg/L) [50].

Table S1. Chemical properties, molecular characteristics, and toxicological parameters of synthetic musk compounds analyzed in this study (sources: National Institute of Standards and Technology (NIST) Chemistry WebBook and CompTox Chemicals Dashboard v2.5.3, United States Environmental Protection Agency (US EPA)).

Abbreviation (Trade name)	Chemical name	CAS No.	Molecular formula	Molecular weight (g/mol)	Henry's law constant (atm· m^3/mol)	Toxicity	Chemical structure
**Nitro musk**							
MK (Musk ketone)	1-(4-tert-Butyl-2,6-dimethyl3,5-dinitrophenyl)ethanone	81–14–1	$C_{14}H_{18}N_2O_5$	294.3	$1.15\cdot10^{-8}$	0.247 mg/m^3 (DNEL*)	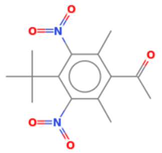
MX (Musk xylene)	1-tert-butyl-3,5-dimethyl2,4,6-trinitrobenzene	81–15–2	$C_{12}H_{15}N_3O_6$	29.26	$1.51\cdot10^{-6}$	$1.50\cdot10^{4}$ mg/kg (LD50**, Rabbit)	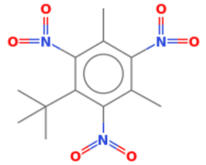
MA (Musk ambrette)	1-tert-butyl-2-methoxy4-methyl-3,5-dinitrobenzene	83–66–9	$C_{12}H_{16}N_2O_5$	268.27	$5.13\cdot10^{-6}$	$2.00\cdot10^{3}$ mg/kg (LD50**, Rabbit)	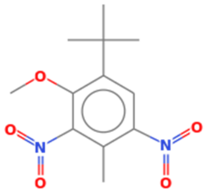
MM (Musk moskene)	1,1,3,3,5-pentamethyl4,6-dinitro-2H-indene	116–66–5	$C_{14}H_{18}N_2O_4$	278.3	$4.68\cdot10^{-6}$	$5.00\cdot10^{3}$ mg/kg (LD50**, Rabbit)	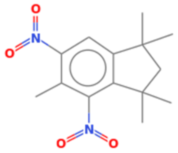

MT (Musk tibetene)	1-tert-butyl-3,4,5-trimethyl2,6-dinitrobenzene	145–39–1	$C_{13}H_{18}N_2O_4$	266.29	$1.15 \cdot 10^{-5}$	$5.00 \cdot 10^{3}$ (LD50**; Rabbit)	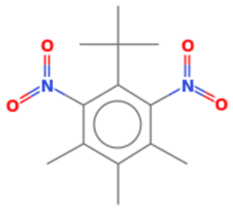
**Polycyclic musk**							
HHCB (Galaxolide)	4,6,6,7,8,8-hexamethyl-1,3,4,7-tetrahydrocyclopenta[g] isochromene	1222–05–5	$C_{18}H_{26}O$	258.40	$3.02 \cdot 10^{-4}$	13.5 mg/m^{3} (DNEL*)	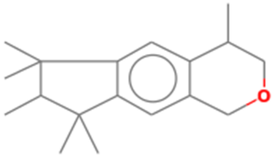
AHTN (Tonalid)	1-(5,6,7,8-tetrahydro-3,5,5,6,8,8-hexamethyl-2-naphthyl)ethan-1-one	21145–77–7	$C_{18}H_{26}O$	258.40	$4.79 \cdot 10^{-5}$	$5.00 \cdot 10^{3}$ mg/kg (LD50**, Rabbit)	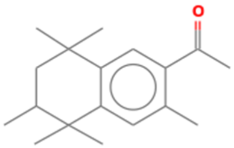
ABDI (Celestolide)	1-(6-tert-butyl-1,1-dimethyl2,3-dihydroinden-4-yl)-ethanone	13171–00–1	$C_{17}H_{24}O$	244.37	$2.09 \cdot 10^{-5}$	-	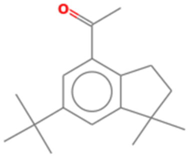
AHDI (Phantolide)	1-(1,1,2,3,3,6-hexamethyl-2Hinden-5-yl)ethanone 1	15323–35–0	$C_{17}H_{24}O$	244.37	$2.04 \cdot 10^{-5}$	$1.70 \cdot 10^{3}$ mg/kg (LD50**, Rabbit)	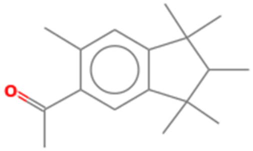
DPMI (Cashmeran)	1,1,2,3,3-pentamethyl2,5,6,7-tetrahydroinden-4-one	33704–61–9	$C_{14}H_{22}O$	206.32	$2.57 \cdot 10^{-4}$	1.47 mg/m^{3} (DNEL*)	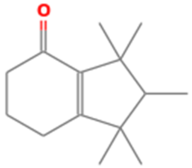

ATII (Traesolide)	1-(1,1,2,6-tetramethyl-3-propan2-yl-2,3-dihydroinden-5-yl) ethanone	68140–48–7	$C_{18}H_{26}O$	258.40	$4.79 \cdot 10^{-5}$	–	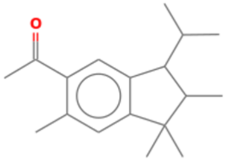
**Other fragrance**							
OTNE (Iso E Super)	1-(2,3,8,8-tetramethyl-1,3,4,5,6,7-hexahydronaphthalen-2-yl) ethanone	54464–57–2	$C_{16}H_{26}O$	234.38	$1.95 \cdot 10^{-4}$	–	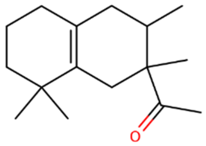

[*] Derived No-Effect Level

[**] Lethal Dose 50%

Table S2. Sampling site information including coordinates, location descriptions, distances from wastewater treatment centers, and total sample numbers.

Sampling sites	Latitude	Longitude	Descriptions	Distance from Water Reclamation Center (km)		Total number of samples
				Nanji	Seonam	
St.01	37°34'14.2"N	126°51'33.6"E	Downstream of the Han River	2.09	3.59	18
St.02	37°36'36.1"N	126°47'50.6"E	Downstream of the Han River	4.95	3.58	18
St.03	37°41'48.0"N	126°39'56.8"E	Downstream of the Han River, Front of Jeollyu-ri Port	20.0	18.6	18
St.04	37°43'08.1"N	126°31'12.5"E	Near Ganghwa-do, Near Ganghwa bridge	32.2	30.6	18
St.05	37°45'22.7"N	126°19'04.3"E	Near Ganghwa-do, Northern tip of Seokmodo	50.1	48.4	18
St.06	37°41'39.5"N	126°14'51.4"E	Near Ganghwa-do, Near Boreumdo	54.0	52.2	18
St.07	37°33'25.2"N	126°25'27.9"E	Incheon coastal area, Near Sindo	37.1	35.3	18
St.08	37°33'20.9"N	126°34'49.9"E	Incheon coastal area, Near Yeongjong Bridge	23.4	21.7	18
St.09	37°29'18.3"N	126°35'41.8"E	Incheon coastal area, Near Incheon North Harbo	24.4	22.8	18
St.10	37°25'18.8"N	126°33'23.5"E	Incheon coastal area, Near Incheon Bridge	31.1	29.7	18
St.11	37°15'15.7"N	126°08'52.3"E	Deokjeokdo	71.7	70.1	18
Nanji	37°35'04.5"N	126°50'36.3"E	Downstream of the Han River, In front of Nanji Water Reclamation Center outlet			12
Seosam	37°35'05.9"N	126°49'22.4"E	Downstream of the Han River, In front of Seonam Water Reclamation Center outlet			12
			Total sample number			222

Table S3. Mean values of selected water quality parameters and total concentrations of SMC by sampling station and depth layer (surface/bottom) during 2021–2022.

Station	layer	Transp-arency (m)	Temp. (℃)	Salinity (psu)	pH	DO (mg/L)	COD (mg/L)	TOC (mg/L)	SS (mg/L)	Chl.a (μg/L)	TN (mg/L)	TP (mg/L)	NH_4-N (mg/L)	NO_2-N (mg/L)	NO_3-N (mg/L)	DIN (mg/L)	PO_4-P (mg/L)	SMCs (μg/L)
St.01	Bottom	1.00	16.40	3.92	7.32	7.36	3.65	2.49	14.47	13.38	3.71	0.13	0.27	0.06	3.13	3.47	0.03	2.24
	Surface	1.00	16.57	3.93	7.34	8.29	3.64	2.87	12.00	12.81	3.75	0.12	0.27	0.06	3.17	3.50	0.03	3.13
St.02	Bottom	1.33	16.45	4.00	7.26	7.21	4.41	2.75	16.48	11.98	4.83	0.14	0.46	0.06	3.70	4.22	0.04	3.90
	Surface	1.33	16.82	4.00	7.22	8.33	4.80	2.93	16.39	15.18	4.78	0.13	0.41	0.06	3.86	4.34	0.03	3.63
St.03	Bottom	0.43	16.23	4.22	7.57	7.55	5.75	2.83	115.43	30.85	4.69	0.20	0.31	0.09	3.78	4.19	0.03	5.03
	Surface	0.43	16.18	4.08	7.55	8.40	5.90	2.90	67.25	30.12	4.57	0.12	0.29	0.10	3.23	3.62	0.03	3.06
St.04	Bottom	0.45	15.60	16.43	7.70	6.82	2.72	2.23	75.28	3.71	2.41	0.12	0.08	0.04	1.41	1.53	0.04	0.82
	Surface	0.45	15.60	15.97	7.66	7.56	2.73	2.06	69.38	3.10	2.43	0.12	0.08	0.04	1.47	1.58	0.04	1.31
St.05	Bottom	0.92	18.13	23.67	7.89	7.32	2.66	1.97	70.42	5.17	1.53	0.13	0.03	0.02	0.79	0.84	0.03	0.25
	Surface	0.92	17.52	22.48	7.87	7.78	2.58	2.29	35.09	8.86	1.72	0.12	0.03	0.02	0.81	0.86	0.03	0.64
St.06	Bottom	1.38	18.62	26.30	7.91	7.88	2.81	1.97	29.65	3.38	0.70	0.07	0.02	0.01	0.42	0.46	0.02	0.07
	Surface	1.38	18.60	25.53	7.89	8.64	2.35	2.03	30.12	5.60	0.76	0.07	0.02	0.01	0.42	0.45	0.02	0.43
St.07	Bottom	0.70	18.57	27.60	7.98	7.40	2.95	2.59	53.30	5.26	0.85	0.09	0.05	0.03	0.52	0.60	0.03	0.61
	Surface	0.70	18.33	27.48	7.98	7.55	3.01	2.06	52.84	3.68	0.81	0.08	0.04	0.03	0.48	0.54	0.03	0.76
St.08	Bottom	1.35	19.57	27.07	7.96	6.80	2.67	2.16	22.86	1.97	0.82	0.08	0.13	0.04	0.40	0.57	0.04	0.19
	Surface	1.35	17.98	26.07	7.90	7.65	2.43	1.97	19.39	2.58	1.04	0.08	0.08	0.04	0.59	0.71	0.04	0.29
St.09	Bottom	1.28	19.42	28.83	8.09	6.96	2.27	2.48	37.04	2.05	0.62	0.09	0.07	0.03	0.23	0.34	0.04	0.07
	Surface	1.28	18.83	28.67	8.08	7.38	2.52	2.21	26.71	2.41	0.74	0.07	0.09	0.03	0.31	0.43	0.04	0.42
St.10	Bottom	1.27	19.33	29.48	8.13	7.36	1.90	1.96	28.47	2.80	0.53	0.09	0.08	0.03	0.24	0.34	0.03	0.19
	Surface	1.27	18.93	29.40	8.12	8.21	2.41	2.79	26.41	2.83	0.58	0.06	0.09	0.03	0.27	0.38	0.03	0.34
St.11	Bottom	2.12	18.80	30.32	8.07	7.50	1.77	1.82	30.99	5.29	0.29	0.05	0.01	0.00	0.08	0.10	0.01	0.41
	Surface	2.26	18.66	30.10	8.05	8.16	1.69	1.72	24.37	2.75	0.30	0.04	0.02	0.00	0.06	0.09	0.01	0.21

**Seonam**	**Bottom**	0.60	22.87	0.27	6.01	6.29	41.26
	**Surface**	0.60	24.15	0.25	6.23	8.10	35.95
**Nanji**	**Bottom**	0.90	22.27	0.27	6.39	35.11	26.60
	**Surface**	0.90	23.85	0.30	6.10	8.93	26.03

Table S4. Pearson correlation coefficients between SMC concentrations and water quality parameters across different sampling periods (2021–2022) and depths (surface and bottom layers). Asterisks denote statistical significance ($^*p < 0.05$, $^{**}p < 0.01$)

	**2021-05**		**2021-08**		**2021-10**		**2022-05**		**2022-08**		**2022-10**	
	**Bottom**	**Surface**	**Bottom**	**Surface**	**Bottom**	**Surface**	**Bottom**	**Surface**	**Bottom**	**Surface**	**Bottom**	**Surface**
**Transparency (m)**	0.10	0.53	0.58	0.32	-0.77	0.00	-0.59	-0.45	-0.35	-0.35	0.18	0.18
**Temp (℃)**	0.67*	0.80**	0.55	0.78**	-0.15	-0.08	0.66*	0.79**	-0.59	-0.67*	-0.36	-0.5
**Salinity (psu)**	-0.83**	-0.89**	-0.79**	-0.82**	-0.76*	-0.64*	-0.75*	-0.78**	-0.53	-0.33	-0.90**	-0.88**
**pH**	-0.18	-0.34	-0.90**	-0.97**	-0.59	-0.17	-0.81**	-0.74*	-0.36	-0.18	-0.82**	-0.74*
**DO (mg/L)**	0.02	0.31	-0.61	-0.25	0.86**	0.02	0.12	0.04	0.37	0.51	-0.05	0.65
**COD (mg/L)**	0.36	-0.02	0.47	0.68*	0.83**	0.45	0.70*	0.47	0.41	0.08	0.33	0.44
**TOC (mg/L)**	0.82**	0.88**	0.74*	0.56	0.17	0.65*	0.03	0.20	0.30	0.07	0.98**	0.98**
**SS (mg/L)**	-0.43	-0.57	-0.08	-0.10	0.73*	-0.18	0.57	0.33	0.46	0.06	-0.46	-0.52
**Chl-a (μg/L)**	0.89**	0.87**	0.21	0.22	0.76*	0.56	-0.18	-0.03	0.29	0.31	0.94**	0.97**
**NH_4-N (mg/L)**	0.89**	0.80**	0.92**	0.85**	0.71*	0.69*	0.84**	0.73*	0.89**	0.59	0.53	0.56
**NO_2-N(mg/L)**	0.84**	0.85**	0.45	0.44	-0.08	-0.21	0.80**	0.69*	0.21	0.68*	0.88**	0.84**
**NO_3-N (mg/L)**	0.81**	0.92**	0.79**	0.83**	0.77**	0.66*	0.87**	0.71*	0.57	0.62	0.93**	0.92**
**DIN (mg/L)**	0.84**	0.92**	0.88**	0.87**	0.77**	0.66*	0.88**	0.73*	0.60*	0.64	0.93**	0.93**
**PO_4-P (mg/L)**	-0.07	-0.27	0.74*	0.65*	-0.64*	-0.55	0.50	0.48	-0.21	0.18	-0.89**	-0.85**
**TN (mg/L)**	0.83**	0.93**	0.89**	0.75*	0.81**	0.71*	0.88**	0.70*	0.66*	0.67*	0.94**	0.95**
**TP (mg/L)**	0.59	0.52	0.59	0.96**	0.35	-0.23	0.29	-0.42	0.42	0.51	-0.32	-0.00

Table S5. XGBoost-SHAP analysis results for environmental parameters affecting SMCs concentrations.

Feature	Mean Absolute SHAP (log µg/L)	Mean SHAP (log µg/L)	Standard deviation of SHAP (log µg/L)	Feature Importance
**TN**	3.2E-01	5.6E-02	3.8E-01	3.6E-01
**TP**	7.4E-02	1.5E-02	8.7E-02	1.3E-01
**DO**	5.3E-02	3.3E-02	6.0E-02	1.2E-01
**TOC**	5.0E-02	6.5E-03	5.8E-02	8.8E-02
**Chl.a**	5.0E-02	-8.3E-03	7.6E-02	1.0E-01
**COD**	3.7E-02	8.9E-03	4.2E-02	9.0E-02
**SS**	3.1E-02	7.1E-03	4.1E-02	1.1E-01

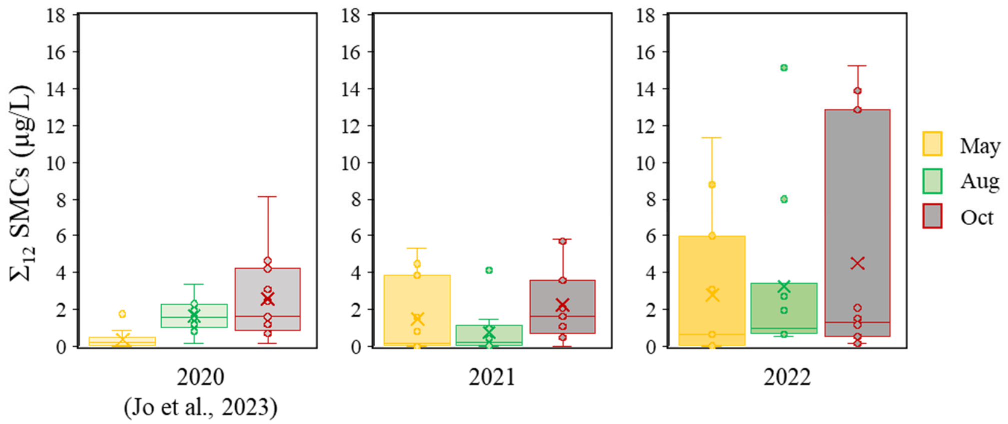

Figure S1. Temporal variation of Σ12 SMCs concentrations in the Han River estuary and coastal waters from 2020 to 2022 (excluding water reclamation centers). Data for 2020 from Jo et al. [37].
